# Transposable Elements Adaptive Role in Genome Plasticity, Pathogenicity and Evolution in Fungal Phytopathogens

**DOI:** 10.3390/ijms20143597

**Published:** 2019-07-23

**Authors:** Nurhani Mat Razali, Boon Huat Cheah, Kalaivani Nadarajah

**Affiliations:** 1 School of Environmental and Natural Resource Sciences, Faculty of Science and Technology, Universiti Kebangsaan Malaysia, Bangi 43600, Malaysia; 2 School of Biosciences and Biotechnology, Faculty of Science and Technology, Universiti Kebangsaan Malaysia, Bangi 43600, Malaysia; 3 Department of Agronomy, National Taiwan University, No. 1, Sec. 4, Roosevelt Road, Taipei 10617, Taiwan

**Keywords:** genome plasticity, evolution, pathogenicity, transposable elements, phytopathogen

## Abstract

Transposable elements (TEs) are agents of genetic variability in phytopathogens as they are a source of adaptive evolution through genome diversification. Although many studies have uncovered information on TEs, the exact mechanism behind TE-induced changes within the genome remains poorly understood. Furthermore, convergent trends towards bigger genomes, emergence of novel genes and gain or loss of genes implicate a TE-regulated genome plasticity of fungal phytopathogens. TEs are able to alter gene expression by revamping the *cis*-regulatory elements or recruiting epigenetic control. Recent findings show that TEs recruit epigenetic control on the expression of effector genes as part of the coordinated infection strategy. In addition to genome plasticity and diversity, fungal pathogenicity is an area of economic concern. A survey of TE distribution suggests that their proximity to pathogenicity genes TEs may act as sites for emergence of novel pathogenicity factors via nucleotide changes and expansion or reduction of the gene family. Through a systematic survey of literature, we were able to conclude that the role of TEs in fungi is wide: ranging from genome plasticity, pathogenicity to adaptive behavior in evolution. This review also identifies the gaps in knowledge that requires further elucidation for a better understanding of TEs’ contribution to genome architecture and versatility.

## 1. Introduction

Sustainable production of crops is crucial to safeguard food security and maintain socioeconomic stability. However, crop production is constantly challenged by biotic and abiotic stresses that threaten sustainable food production. Diseases such as blast, (rice), stem rust (wheat) and corn smut (maize), are main contributors to yield losses in many agriculturally dependent countries [1]. Major pathogens that cause losses in the field are bacteria, fungi and viruses. These pathogens are rapidly evolving, leading to breakdown in resistance to these microorganisms in crops [2,3]. One interesting microorganism is fungus. Fungal diseases are rather difficult to control as the spores disperse easily and their presence in soil and air persist despite the application of chemical controls. While breeding for resistance through quantitative trait locus (QTL) identification [4,5,6], application of fungicide and improved agricultural practices are current methods utilized to control plant diseases, the understanding of the fungal pathogen itself is crucial in establishing effective defense. 

Understanding fungal pathogen lifestyles and mechanisms of evolution is crucial in controlling the spread of fungal diseases. One sure way of understanding the organism is by unravelling the regulatory mechanisms underlying the molecular composition of the fungi. This will provide us with the understanding of the adaptive nature of these organisms, their mechanism of survival and pathogenicity. The advent of the genomic era has resulted in a surge in the availability of microbial genome sequences, including that of the destructive fungal phytopathogens [7]. Through genome information, characterization at the molecular level of fungal phytopathogens has enabled researchers to unravel their lifestyle [8,9]. One such molecular component that has been implicated in the adaptive nature of fungi are the transposable elements (TEs). The abundance of TEs in eukaryotic genomes [10] and their ability to cause genome restructuring has garnered interest from the research community. Previous studies on TEs in fungal phytopathogens have reported their influence on genome plasticity [11,12], pathogenicity [13,14], host range [15] and evolution [16,17]. These influences are mediated through mutations, chromosomal reorganization, altered expression, and the generation of new proteins associated with TE insertions. In this regard, it is important to decipher TE-mediated effects on fungi in the destruction of economically important host species. Therefore, in this review we have looked into the complex roles played by TEs in phytopathogenic fungal genome structure, rearrangement, and their contribution to the pathogenic lifestyle. The gaps in knowledge have also been highlighted.

## 2. Transposable Elements (TEs) in Brief

A brief overview on TEs will provide the information on the roles played by these elements in genomes. Since the discovery of TEs in the mid-1940s [18], perception of TEs has changed from being just junk DNA to a major contributor of genomic variation. TEs are present in virtually every prokaryotic and eukaryotic genome. Despite their abundance in genomes, only a few TEs have retained their mobility while the rest are no longer mobile. TEs can be autonomous or non-autonomous, where the former possesses genes encoding transposing enzymes and the latter can be defective from the lack of genes encoding the enzyme to transpose. Occasionally, the non-autonomous TEs can transpose via genes encoding transposase located elsewhere [19]. Their mobile nature has also resulted in these elements being referred to as jumping genes. By inserting themselves into protein-coding genes, TEs can affect genes [20] by either providing novel regulatory sequences [21] or via epigenetic silencing of TEs [22] or through the mutation of the interrupted gene [23]. TEs can also cause mutation in a larger scale through chromosomal rearrangements such as deletions, duplications, inversions and translocations through homologous recombination and alternative transposition [24]. While most TE activity is generally deleterious to organismal function, some insertions may have adaptive importance to the host. Besides providing adaptive traits to the host genome, TEs have roles in cellular function as exhibited by TEs found in the centromeres. TEs can be found in inordinate amounts at different regions of the chromosome including genic, pericentric, centromeric, or even telomeric regions, which may vary according to the species. There has been extensive discussion on TEs’ possible role in the evolution of centromeres function and structure. Previous studies have shown that TEs contribute in conferring centromere stability, kinetochore assembly, centromere formation and centromeric function through TE domestication [25,26,27,28,29]. However, the contribution of TEs to centromeres in fungal phytopathogens is poorly discussed due to the lack of studies in this aspect. 

The first TE classification system was proposed by Finnegan [30] in 1989 where TEs were classified into two classes based on their transposition intermediate: RNA (class I or retrotransposons) or DNA (class II or DNA transposons). In this system, Class I elements employ a “copy-and-paste” mechanism while the Class II elements utilize a “cut-and-paste” mechanism. However, the discovery of bacteria and eukaryotic TEs that exhibit the “copy-and-paste” transposition mechanism without the involvement of RNA intermediates has challenged this system and led to the proposal of TE classification systems by Wicker [31], Repbase [32] and Curcio and Derbyshire [33]. Piégu et al., (2015) reviewed the advantages and weaknesses of these improved TE classification proposals [34]. The following discussion on Class I and Class II elements will be based on Wicker’s proposal. 

**Figure 1:**
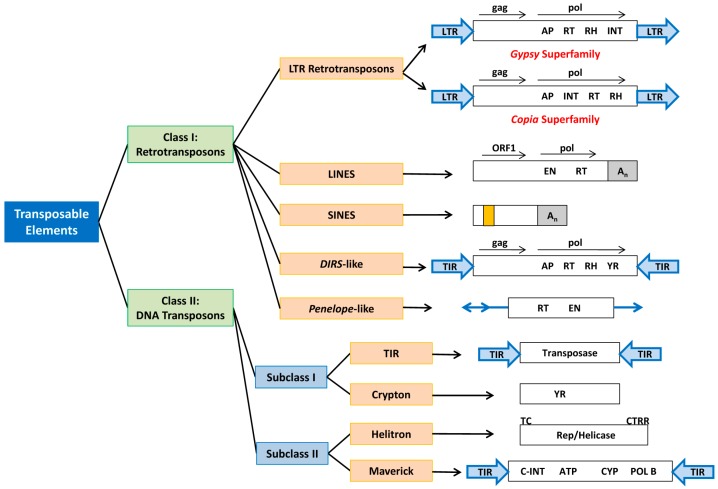
The orders and superfamilies of transposable elements present in fungi. (Note: LTR = long terminal repeats, TIR = terminal inverted repeats, AP = aspartic protease, RT = reverse transcriptase, RH = RNase H, INT = integrase, EN = endonuclease, An = poly(A) tail, YR = tyrosine recombinase, ATP = packaging ATPase, CYP = cysteine protease, POL B = DNA polymerase B. Internal RNA polymerase III promoter region of SINEs is indicated by orange box.) (Source: Credit Ilakiya Kumar).

In Wicker’s proposal, TEs are hierarchically classified into class, subclass, order, superfamily, family and subfamily (Table 1; Figure 1). Similar to Finnegan’s classification system, Wicker’s proposal also divides TEs into two classes based on the presence or absence of a RNA transposition intermediates while the subclass functions to distinguish between the “cut-and-paste” and the “copy-and-paste” DNA transposition mechanisms. Furthermore, class I and class II TEs with different encoded enzymes are classified under separate orders. TEs from different superfamilies share similar insertion mechanisms but have different enzyme organization, non-coding domains and/or target site duplications (TSD). Table 1 below summarizes each hierarchical level described by Wicker’s classification [31]. Class I TEs transpose via an RNA intermediate that is reverse transcribed into cDNA before it is integrated into another genomic locus. Therefore, a new TE copy is produced in each transposition cycle and hence it is called the “copy-and-paste” mechanism which is able to increase copy numbers by transposing from one genomic site to another [35]. Class I TEs can be divided into five orders based on their insertion mechanisms: long terminal repeat (LTR) retrotransposons, dictyostelium intermediate repeat sequence (DIRS)-like elements, Penelope-like elements (PLEs), long interspersed nuclear elements (LINEs) and short interspersed nuclear elements (SINEs) [19,31] (Figure 1).

Class II TEs transpose via DNA intermediate and are divided into subclass I (“cut-and-paste”) and subclass II (“copy-and-paste”). Subclass I TEs are divided into two orders namely terminal inverted repeats (TIR) and Crypton whereas subclass II TEs are divided into Helitron and Maverick orders [31] (Figure 1). Transposase of the TIR order is responsible for active transposition where two transposases will bind to the flanking TIR sequences for excision [36]. The new TE insertion is flanked by a small gap at the insertion site that will be filled in by host enzymes to form TSD. Based on similarity of their transposases, TIR sequences, and TSD size, this order has been classified into nine superfamilies (Tc1-Mariner, hAT, Mutator, Merlin, Transib, P, PiggyBac, PIF-Harbinger and CACTA) [31,37]. 

## 3. TEs: Adaptive Drivers of Evolution

**Figure 2:**
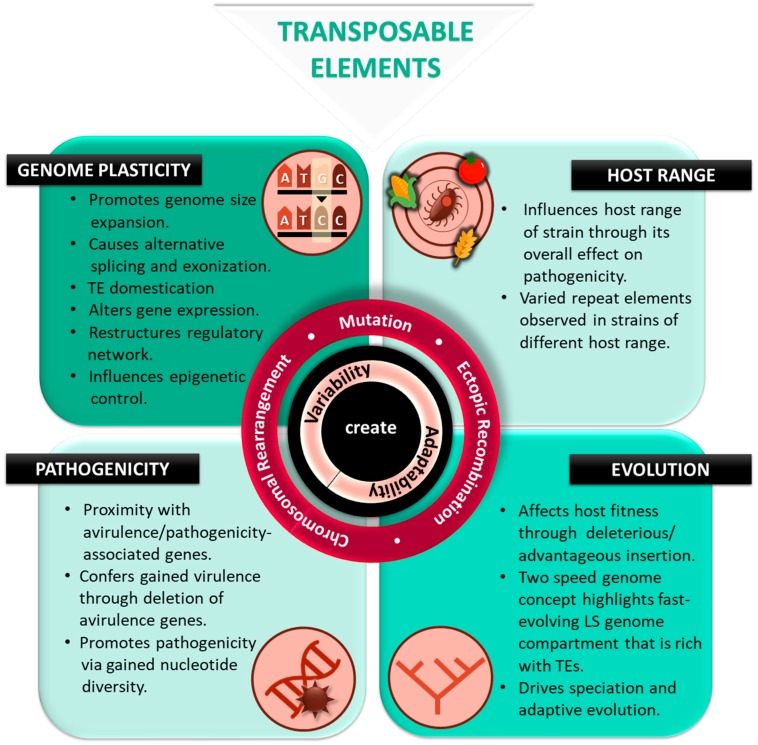
The role of transposable elements in affecting genome plasticity, influencing host range and pathogenicity, and shaping evolution of phytopathogens. Transposable elements generate genetic variability that can contribute to host adaptation to its surroundings. The mechanisms involved include TE-mediated mutation, ectopic recombination and chromosomal rearrangement. Collectively, TEs can influence agriculturally relevant traits exemplified by observations made in rice pathogens and other fungal phytopathogens. (Source: Credit Ilakiya Kumar).

Genome plasticity enables organisms to adapt to environmental changes and occupy novel niches. Adaptive evolution mediated by TEs is facilitated by recombination events resulting in genomic diversification. This is achieved through genomic changes, which persist under positive selection in fungal pathogens. TEs contribute towards adaptive genetic variation through: (1) TE insertion into coding genes [26], (2) TE insertion into introns, (3) TE transposition in proximity to genes, (4) generation of retrocopies via reverse transcription [38], and (5) aberrant transposition and ectopic recombination through paralogous TEs [39]. Adaptive changes in the genome are subject to natural selection where deleterious insertion that affects host fitness will not be retained in the genome (Figure 2) while, advantageous and neutral insertions will be maintained and vertically transmitted. The survival of TEs in a given genome environment can be promoted through the propagation of TE copies via vertical transmission, horizontal transfer and intra genomic transposition [40]. TEs are also known to multiply and evolve independently within the genome, where TEs that produce more copies have a higher capacity to invade genomes. As a longstanding element within the genome, TEs are recruited by the host genome for molecular domestication leading to the formation of TE-derived genes and are recognized as players in the arm’s race between host-pathogen [41]. 

Möller and Stukenbrock, (2017) on the other hand described fungal phytopathogen evolution as managed versus natural ecosystem and ‘trench warfare’ versus ‘arm race’. The observed trend is that fungi have evolved rapidly and are highly adaptive to new environments. However, TE density does not directly correlate to a rapid rate of evolution as genomes with low TE content can exhibit rapid evolution [42]. Through co-evolution between pathogen and host, TEs influence adaptability which is beneficial towards the survival of the fungal pathogens [36,43]. Seidl and Thomma, (2017) described fitting roles for TEs in the co-evolution between plants and their associated microbes where TEs mediate the adaptation of plants through the immune system while the predatory microbe acclimatizes through effector action. This is achieved by alteration in gene expression and structural changes [44]. 

Furthermore, TEs’ remarkable role in adaptive evolution can be elucidated through the two-speed genome concept of pathogen evolution (Figure 2). This concept highlights the division of genome architecture into two compartments. One compartment evolves slowly, lacks repeat elements, gene-rich and contains core genes responsible for basic physiology. The other compartment evolves rapidly with abundant repeat elements and poor gene content. This compartment serves to drive adaptive evolution where it houses effector genes involved in pathogenicity [45]. Effectors are virulence factors that weaken plant immunity and are highly expressed during the infection of the host [46]. The genes harbored in this region will favor niche adaptation, accelerated evolution and survivability of the pathogen [17,41,47,48]. This is also seen in the lineage-specific (LS) region that drives adaptive evolution. The improvement in the genome assembly quality and identification of TEs in *Magnaporthe oryzae* through PacBio sequencing allows for scrutiny of a lineage-specific [49] region that harbors high repeat content and is more plastic. In addition, larger proportions of secreted proteins are found in the LS region in comparison to core regions [50] as observed in *Verticillium dahliae*, a broad-range fungal plant pathogen causing vascular wilt disease. This region is rich in active TEs and also harbors effector genes, including *Ave1* that is significant for host recognition in initiating virulence [48]. Furthermore, TEs enriched near synteny breakpoints are shown to facilitate homology-based recombination. The presence of active TEs in the fast-evolving compartment of LS region contributes towards the plasticity of the genome and potentially the pathogenicity of the fungal pathogen [48]. However, there is also an exception to the “two-speed genome concept” as observed in the filamentous phytopathogen, *Blumeria graminis f. sp hordei* (Bgh), a powdery mildews fungus that employs a particularly high-speed, one-speed genome. TEs that are evenly distributed in the Bgh genome are mostly associated with coding regions of various biological processes including avirulence genes. TEs occupy most of the Bgh genome and the genome is gene poor but TE-rich throughout [51].

TEs’ mediated impact on the fungal pathogen adaptability to new environment and overcoming host resistance is presented in the following examples. Ma et al. (2010) reported that *Fusarium oxysporum* adaptation to a new host is facilitated by TE-rich insertions in the ‘B’ chromosomes [52]. Broadening of the pathogen host range via lateral transfer of TE-enriched pathogenicity gene clustered chromosome has also been reported in *Alternaria* sp [53]. TE-mediated mutations drive speciation through new niche exploration and increase in fitness level (Figure 2). Progress in pathogen evolution is steered by the success in overcoming host immunity, developed resistance and the capacity to ‘jump’ from host to host [47]. For instance, the emergence of *M. oryzae* is a consequence of host shift in Setaria millet, following the opportunistic timeline of rice domestication. Host shift is often promoted in new species that are highly important, widely distributed and with high-density population such as rice. The host shift is founded on the expansion of TE copy number, MGR586 (Pot3), followed by the loss of a specific avirulence gene, *AVR-co39*, that allows for the jump to new host. The loss of avirulence gene indicates that the AVR-R gene interaction may prevent the initiation process of host shift hence resulting in subsequent inability to invade rice. Hence, TEs have a big influence on host specialization by promoting the loss of avirulence genes and the breakdown of complete resistance, based on rare-specific R genes [54,55].

A study involving 62 strains of *M. oryzae* on 6 Avr-genes indicate the significant presence or absence in polymorphism and a high ratio of non-synonymous to synonymous substitution which may be the underlying mechanism used to evade the host plant system through rapid adaptive evolution. Interestingly, repeat sequences including TEs are found flanking most of these reported Avr-genes [56]. Furthermore, the clonal population of *M. oryzae* and *F. oxysporum* reported mutations in their repetitive genome compartments [57,58,59]. From reports of asexual speciation of *Verticillium dahliae* and *F. oxysporum*, we stipulate that recombination may contribute towards adaptive evolution in a primarily clonal community. This observation is further substantiated by reports that *M. oryzae* and *Puccinia striiformis* formae speciales (f. sp.) *tritici* have evolved towards generation of asexual clonal linages with ferocious spreading abilities. In addition, high TE activity in *Aspergillus oryzae* [10] and *M. oryzae* [13] is hypothesized to be the reason behind the rapid strain evolution in the field.

Therefore, to maintain a pathogenic lifestyle, adaptation to host and environment is essential. We hypothesize ecological adaptation to host and environment may be achieved through the combinatorial and mutational traits of TE. The TE-induced speciation may promote the emergence of advantageous alleles that are essential in host-pathogen evolution against changing environments, host and fungicide. We believe that if the pathogenicity clusters are maintained under the pressure of TE evolutionary adaptations, the pathogen is then likely to be dynamic in resistance, and adaptation to the current host or new hosts. Nevertheless, host fitness is diminished through non-adaptive and deleterious changes caused by TEs. TEs’ role in adaptive evolution requires further studies in population genetics and extensive analysis of fungal genomes to connect the effect of TEs to mutational fitness.

## 4. TEs: Structural Transformers of the Genome

Genome plasticity can be described as alterations observed in a genome structure, which can be characterized by changes in genome organization, chromosome number and genome size [48]. Genome plasticity is mainly influenced by environmental stresses faced by individual genes in host species that confer genetic adaptability traits for survivability where TEs may play an assistive role [60]. Eukaryotic genome plasticity can be caused by TE-mediated chromosomal rearrangements through ectopic homologous recombination or alternative transposition. Furthermore, the mutation caused by TE insertion may also lead to generation of new proteins promoted by exon shuffling and TE domestication [61]. TE insertions within functional genes may bring about alternative splicing resulting in altered protein synthesis. For instance, a TE insertion within an intron may cause exonization, where the intron with an acquired splice donor and splice acceptor can be alternatively spliced with other exon cassettes [62]. All of these TE insertion-promoted processes, viz. exon shuffling, TE domestication and exonization, can generate novel genes with possible specific functions in the host [36,60] (Figure 2). Meanwhile, if TEs are inserted directly into the coding genes, they disrupt gene function due to altered nucleotide sequences, and affect resulting proteins [60]. 

TEs promote genome size expansion due to its replicative nature where convergence towards a large genome is an observed trend for fungal phytopathogens (Figure 2). Even though this does not include all species, trends in genome expansion can be seen in ancient parasite lineages, such as the ascomycete powdery mildew fungi (64% repetitive of 120 Mbp in *Blumeria graminis* DH14) and the basidiomycete rust fungi (45% repetitive of 220 Mbp in *Melampsora lini* CH5) [47]. The genome size of the causal agent of coffee leaf rust, *Hemileia vastatrix*, is 797 Mbp which is bigger than the 710 Mbp size of its diploid *Coffea canephora* host plant [63,64]. This genome expansion is significantly attributed to repetitive elements including TEs. Furthermore, a study of the 1090 fungal genome sequences in the public repositories revealed that the genomes of 191 phytopathogen species are slightly larger than that of non-phytopathogenic fungi [65]. These studies signify the contribution of TE-mediated genome expansion in host–pathogen interactions where genes encoding for relevant proteins were frequently found in repeat-rich genomic regions [66]. However, this is in contrast with the observed evolutionary trends in parasites and bacterial symbionts where genomic shrinkage is reported [66]. The genome size expansion promoted by TE proliferation, however, may not be the case for all fungal pathogens, where *Ustilago maydis* for example exhibited compacted genome and lack of LTR retrotransposon content [67]. 

TEs’ activities are also significantly linked to the gain or loss of genes where the gain/loss in polymorphism can be observed in the genomes of *M. oryzae* infecting different hosts [15]. For instance, more than 345–441 genes were lost in the *Oryza* Ina168, a *M. oryzae* isolate infecting rice, in comparison with other isolates that infect foxtail, goosegrass, wheat and oat. This observation coincides with the event of host shift and host specialization where some genes were lost through purifying selection and genetic drift [15]. The same supporting observation is noted in *Blumeria graminis* f.sp. hordei, an obligate biotrophic powdery mildew fungus with 64% of its genome made up of TEs. The expansion in retrotransposition activities predominantly from non-LTR groups and the inactivation of repeat-induced point mutations (RIPs) in the obligate biotroph not only increased its genome size but also led to gene losses compared to autotrophic ascomycetes. These missing genes are related to primary and secondary metabolism, plant cell wall depolymerization and toxin secretion which are pathogenesis-associated functions not required in an obligate biotrophic fungus [68]. From the two aforementioned examples of *M. oryzae* and *B. graminis*, we can deduce that TEs not only orchestrates the host specialization of fungal phytopathogens but also influence the adaptability to changing environments.

Another effect from TE insertion is the restructuring of transcriptional and post-transcriptional regulatory networks (Figure 2). TE insertions can give rise to ‘de novo’ genetic networks or restructure existing regulatory networks. ‘De novo’ *cis*-regulatory elements can emerge from TEs that have undergone point mutations [69]. Autonomous TEs depend on host machinery to express their genes for replication, and predispose the evolution of *cis*-regulatory sequences in TEs to mimic the host promoters. For example, in the LTR of mammalian endogenous retrovirus [55,70], the two flanking LTRs in coding regions contain duplicated copies of *cis*-regulatory sequences and RNA Polymerase II promoters [49]. Through ‘de novo’ or restructuring of existing networks, active TEs can produce a variety of regulatory networks to fine-tune gene expression of important biological processes in the phytopathogens. On the other hand, the activity of genomic TEs is counterbalanced by the host genome defenses. One of the genome defense mechanisms is RIPs that are mediated through transcriptional (heterochromatic) silencing of repetitive DNA via cytosine methylation and mutation [22,60]. Through this, TEs indirectly recruit epigenetic control of effector gene expression present in the repetitive genomic regions of the pathogenic fungi (Figure 2). The epigenetic mark, mainly DNA methylation, determines the formation of genomic regions that are either transcriptionally repressed heterochromatin or transcriptionally active euchromatin. The epigenetic mark within a species population was reported to be variable [71] and environmentally influenced [72]. As DNA methylation is dynamic and reversible, this form of TE-mediated genomic plasticity can alter gene expression of fungal phytopathogens spontaneously, especially their interaction with host plants or response to unfavorable environmental conditions [73].

## 5. TEs: Mediators of Pathogenicity and Host Range

Effector proteins that are secreted by fungal pathogens to promote colonization interfere with host defense and result in necrosis. By the same token, effector proteins can be recognized by their complementary plant resistance genes, leading to the activation of defense responses in plants [40]. Pathogenicity genes that are often clustered within a specific region of the genome codes for these molecules that promote infection of host plants. TEs’ recurring association with pathogenicity can be seen by looking at the positional aspect. TEs often sit in proximity with pathogenicity factors as seen in *M. oryzae* where genes that encode secreted proteins are found within 1 kb flanking distance from TEs [50]. Active transposition activities were reported through genome analysis in field isolates of *M. oryzae* Y34 and P131, against the reference genome of *M. oryzae* 70-15. Lack of conserved TE loci between these isolates was observed despite thousands that were found, thus supporting active transposition activities. Notably, most TEs found are in close proximity with duplicated genes and isolate-specific genes, including avirulence genes. The reported duplicated genes with known functions are predicted to be associated with primary and secondary metabolism and host interaction, which is similar to the observations made in isolate 70-15. The transposition of TEs and its association with duplicated genes as well as isolate-specific genes may be the most critical factor in contributing to genome variation in *M. oryzae* [74].

The TE-pathogenicity gene association is also displayed in other fungal pathogens. *Mycosphaerella fijiensis*, a fungus that causes black Sigatoka in Banana has 11.7% of TEs in its genome. A total of 339 protein encoding genes including crucial metabolism and pathogenicity-associated genes were found within 1000 bp upstream from these TEs [75] (Figure 2). Meanwhile, *Mycosphaerella graminicola,* a fungal wheat pathogen that causes *Septoria tritici* blotch, contains approximately 17% repeat elements, mostly Class I TEs (70%). A few genes involved in pathogenicity were in proximity with these nested clusters of TEs. These repeat clusters may act as a ‘breeding ground’ where new pathogenicity determinants can emerge as shown previously in studies of fungi and oomycetes [76] (Figure 2). In studies, involving smut fungal pathogens such as *U. maydis*, *Sporisorium reilianum*, *Ustilago hordei* and *Sporisorium scitamineum*, genes encoding secreted effectors are found pooled in clusters. Notably, these clusters are in the vicinity of repeats, particularly uncharacterized repeats in comparison with other genes, suggesting the need to further characterize these repeats and their contribution to the genome [77]. Further, a review by Möller and Stukenbrock, (2017) highlights how TEs are largely linked to virulence effectors and rapid evolution where most virulence-associated genes are positioned in genome compartments that have abundant TEs and are rapidly-evolving. These regions comprise of non-core chromosomes, clusters of duplicated genes, and AT isochores. It is believed that since there is a strong correlation between pathogenicity gene clusters and TE abundance, the evolution of pathogenicity clusters via TE is likely to result in new strains with the ability to overcome plant immunity [12,19,20,21,30,31,32,33,34,35,36,37,78]. The manifestation of this genome compartmentalization showed a fast-evolving region that provides adaptation to the genome despite the disruptive effect it poses on native genes under a strong diversification selection [77].

TE insertion mediates pathogenicity through mutational effects on pathogenicity-associated genes. Mutations from TE insertions can lead to genetic variability that generates many new pathogenic variants with conferred ability to invade previously resistant host plants (overcome host plant resistance) and thence expand on its host range. TE mediated inactivation or deletion of PAMP-encoding genes or effector genes important for host recognition results in gain of virulence (Figure 2) by evading the plant’s immune system [79]. A genomic region occupying *Avr4/6* in *Phytophthora sojae*, which is responsible for virulence in soybean, revealed a Ty1/Copia-like element in proximity to this locus. This TE insertion caused point mutations conferring virulence to this locus [80].

Variable inter- or intra- species TE abundance can also be potentially linked with a gain of virulence and subsequent expansion of host range. Ma et al., (2010) highlighted the gain of virulence through the presence of mobile pathogenicity chromosomes and its correlation with enriched TE content in LS regions of *F. oxysporum.* TEs are over-represented in the lineage-specific region of *F. oxysporum* (broad host range) with over 74% of its genomic TEs present in the LS regions, an observation not seen in both *F. verticillioides* (narrow host range) and *F. graminearium* (narrow host range). LS region is presumed acquired through horizontal transfer and mobilization of host-specific unique chromosomes into this region. The transfer of these LS chromosomes has been experimentally proven to change non-pathogenic strains to pathogenic strains. In addition, the differences in repetitive sequence content between *Fusarium* species suggest that TEs introduce pathogenicity to new environments by providing genes required for host-specificity [52]. Furthermore, the varying host range among the different anastomosis groups (AGs) in *Rhizoctonia solani* is an area that is worth further studies. The comparative analyses between different AGs namely AG8, AG3 and AG1-1A, highlights differences in SNP and repeat content which may reflect the differences seen in their host range and infection strategies [81] (Figure 2).

TEs role in modulating pathogenicity can also be seen in the regulation of pathogenicity gene expression. Recent findings suggest that the regulation of effector gene expression is present within epigenetically controlled TE-rich regions [82]. The pathogen can evade the plant defense system by temporarily repressing the transcription of effector genes while waiting for the best instance to invade the host by expressing the effector genes. This is achieved through epigenetic control of effectors where repeat-rich regions were reported to show distinct histone methylation that functions to repress the expression of effector genes before host colonization by fungal phytopathogens [46]. In *U. hordei*, TEs positioned upstream of the promoter interferes with the expression of *UhAvr1* avirulence gene through in situ mutation. This results in gain of virulence where the pathogen is able to evade host recognition and thus overcome resistance conferred by resistance gene *Ruh1*, in the host plant. *UhAvr1* is located in effector clusters that are approximately 50% richer in TEs [70].

Collectively, previous findings have shown that TE insertion is able to modulate pathogenicity and host specificity of a fungal pathogen by producing genetic variants of virulence factors to evade recognition by the host plants. This may explain the abundance of TEs found in the vicinity of virulence clusters. The abundance and proximity of TEs to the effectors clusters harbored in fast-evolving genome compartments may be a preferred strategy and a selective advantage of the fungal pathogen to compete in the co-evolutionary arms race with the host plant and thence promote its pathogenic lifestyle. Both plant and pathogen are subject to active evolutionary changes, especially in modern agricultural settings where breeding programs have introduced resistant cultivars that present strong selection pressure [42,77]. Therefore, it is worth exploring if TE and virulence factor proximity is a consistent observation for fungal pathogens inter- and intra-species and if this abundance may be exploited by fungal pathogens to expand their host range.

## 6. Conclusions

Over decades, the perspectives of TEs in fungal genomes have shifted. TEs’ presence and activities promote two main traits; variability and adaptability. TE insertion promotes genomic variability that may lead to generation of new proteins that confer possible advantages to fungal survival. In fungal phytopathogens, their survival lies in overcoming the plant defense system and hence initiating infection. These are often accompanied by TE-mediated loss of avirulence genes and diversification of pathogenicity genes as shown in *M. oryzae*. A number of studies have shown the proximate of TEs to pathogenicity-associated genes. This trend of genes present in proximity to TEs is worth exploring to see the possibility of preferential insertion. As described in earlier sections, most fungal phytopathogen subscribe to a “two-speed genome” where the genome is divided into two compartments. The rapidly evolving and repeats rich region called lineage-specific [43] is the cradle for adaptive evolution. However, there may be exception to the above.

A deeper look into the effects of TEs in agriculturally relevant traits such as pathogenicity, host range, and genome plasticity will be useful to provide molecular perspectives on the driving factors of virulence to hosts. Nonetheless, there are still unexplored gaps in the study of TEs that leaves room for more interpretational analyses pertaining to TE traits and the possible impact on phytopathogens as follows:

1. What are the properties of TEs that may be implicated in pathogenicity?
2. What is the correlation (positive or negative) between TE abundance and the spectrum of host range (narrow, intermediate, broad) in a phytopathogenic fungi?
3. Are TE activities and its presence random in nature or preferentially inserted to alter the genome dynamics in order to suit the needs for genome survival?
4. Can TEs be manipulated to provide translational applications in mitigating crop disease?

Through this review we note that there is no specific trend seen within fungi from the same family or even genus. However, with improving technologies, annotation mechanisms, and the outpouring of genome data, more information may be surmised on the TE’s contribution to host-pathogen interaction and this information may be of potential use in addressing plant diseases. Through in depth studies of population genetics and genome databases, a clearer picture may be obtained on the roles of these elements in fungal phytopathogens and their contribution in their respective lifestyles.

## Figures and Tables

**Table 1:** The hierarchical classification of transposable elements.

Level	Description
Class	It divides transposable elements (TEs) into two classes based on their transposition intermediate: RNA (class I or retrotransposons) or DNA (class II or DNA transposons).
Subclass	It separates TEs that transpose via “copy-and-paste” mechanism from those via “cut-and-paste” mechanism.
Order	It distinguishes TEs with different insertion mechanisms due to dissimilar encoded enzymes.
Superfamily	Superfamilies within an order share the same insertion mechanism but are different in terms of enzyme organization, non-coding domains and/or TSD.
Family	It is defined by DNA sequence conservation.
Subfamily	It is defined on the basis of phylogenetic data and might serve to differentiate autonomous and non-autonomous derivatives.
